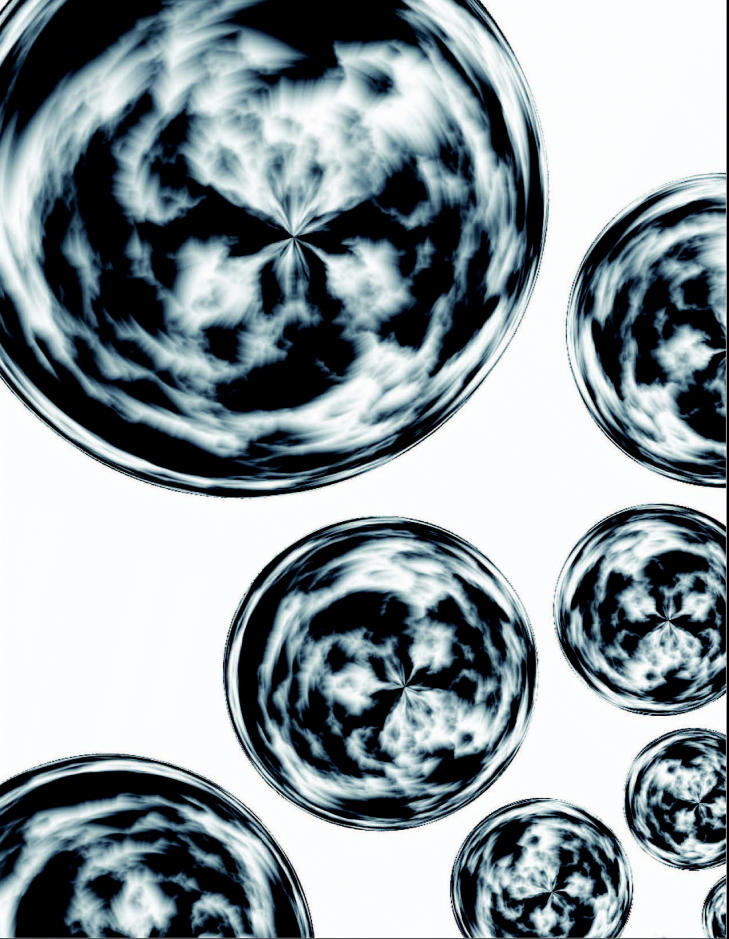# Fluid Movement

**DOI:** 10.1289/ehp.114-a710

**Published:** 2006-12

**Authors:** John Manuel

Each Christmas, toy stores sell music featuring miniature skaters boxes that magically circle and spin around a mirrored surface. These figures are controlled by magnets hidden beneath the “ice.” That same principle is being used by scientists at Arizona State University (ASU) along with partners at the Universidad Nacional de Educacion por Distancia in Madrid, Spain, to manipulate tiny drops of fluid for purposes of analysis. Discrete magnetic microfluidic technology, which is being developed primarily at ASU, promises dramatic reductions in cost and increases in speed and accuracy of analyzing fluids, with positive implications for the field of environmental health.

## Learning in a Liquid Environment

Microfluidics is an emerging field that involves the design, manufacture, and formulation of devices that deal with volumes of fluid on the order of nanoliters or picoliters. There are many benefits in dealing with such tiny amounts of fluid, primary among these being speed and flexibility in analysis. Water, blood, bacterial cell suspensions, and protein or antibody solutions can all be analyzed using microfluidic devices. Applications for microfluidics include immunoassays, DNA analysis, cell manipulation, and analysis of proteins via mass spectrometry.

In performing chemical analyses with microfluidic devices, sample drops of fluid are typically combined with a reagent and channeled to some sort of sensor for detection of a target substance. But as the channels become smaller and narrower, their confining walls pose a problem because of their propensity to capture or denature proteins. This contaminates the sample and makes it difficult to conduct quantitative measurements or run multiple samples through the same device. This, in turn, limits the accuracy of detection.

“Contamination is a major problem in drug testing,” says Mark Hayes, a professor of biochemistry at ASU. “Chemical compounds on the surface want to stick to and be a part of the next sample.”

One of the challenges in microfluidics is thus to find ways to minimize the impact of device walls. Newer technologies involving fluid encapsulation in droplets eliminate the limitations of the device walls while introducing new challenges for the manipulation of droplets. These challenges include the controlled merger of droplets, the splitting of droplets, looking inside of droplets, mixing inside of droplets, storing droplets, and sorting droplets.

The ASU team has developed a unique superhydrophobic surface, across which beads of fluid can be moved with ease. The surface is flat, and embedded with nanowires approximately 40 nm in width and approximately 2,000 nm in length. Fluids are repelled by these needles as there is no edge for the drops to grab onto.

Paramagnetic particles (which display magnetic properties only in the presence of an external magnetic field) are placed on the superhydrophobic surface with a drop of fluid on top, allowing the two to mix. A magnetic field is generated by use of a bar magnet located below the surface. Droplets containing paramagnetic particles are moved around the surface by activation of the magnet.

“The magnetized particles inside our droplets form chains that want to get closer to the pole of the magnet,” explains Antonio García, an ASU professor of bioengineering. “They push against the drop, causing it to slide across the surface.”

## The Superhydrophobic Advantage

The combination of a superhydrophobic surface and magnet-driven motion allows for extremely fast and precise manipulation of microlevel amounts of fluids. In studies published in the 17 July 2006 issue of *Applied Physics Letters*, García and colleagues used water drops ranging in volume from 5 to 35 μL. They observed drop movement at speeds of up to 7 cm/sec in both straight and circular paths. “This is more than sufficient to be able to perform any task in microfluidics in under a few seconds,” García says.

Droplets ride across air pockets between the tips of the nanowires and, like a person lying on a bed of nails, never actually come in contact with the surface material other than the nanowires. This minimizes the potential for contamination of fluid samples.

The technology can expand the capability of existing bioassays, separation technologies, and chemical synthesis techniques. García’s team has found that by using two magnetic fields, they can actually rip droplets in two, like chewing gum, and bring them back together. Coalescence and drop splitting are essential processes in microfluidic applications.

“This allows us to combine a drop with an analysis drop, which is useful, for example, in measuring an enzyme,” García says. “The tearing apart allows you to split the sample. You can do this over and over, and retain an even mixture in your fluid.” García says drops can be combined using traditional microfluidic techniques, but splitting is more difficult.

All of these innovations have tremendous implications for the fields of biomedical research, pharmaceuticals, and environmental health. “Think about the ability to take a single droplet and move it to multiple stations in a lab,” Hayes says. “You could conceivably run as many as twenty or thirty tests on a single drop of blood. You could start with a simple question—if X is high, what else do we test for?—and move it on to other stations.”

With the development of microfluidic devices, analyses formerly done in a lab can be done in the field using portable devices and small amounts of fluid, so-called lab on a chip technology. “We can take the lab to the sample rather than the sample to the lab,” García says. “You can save money by reducing the need for lab equipment and personnel, and save time by avoiding the need to transport samples, clean and prepare diagnostic equipment, conduct the analysis, and write up reports. You may be able to get a medical diagnosis of someone’s health condition in fifteen minutes rather than having it take two days.”

## Moving in New Directions

At ASU, García is working with Hayes and Joseph Wang, a professor in the department of chemical engineering, to develop complementary electrical analysis methods and optical strategies to rapidly measure amounts of biochemicals and proteins, primarily in blood. Such methods could be used for rapid diagnosis to identify or rule out conditions like cardiovascular disease.

“We’re trying to take this basic idea of controlling the drop and integrate it with the latest sensor technology,” García says. “We’re thinking of taking the drop and doing a quick separation of proteins with an electric field, then taking those two drops [one containing a reagent and one not] and applying the right sensor to them.” By doing this, he explains, the team can improve the sensors’ ability to detect a broader range of proteins.

Another innovation using discrete magnetic microfluidics involves the ability to move droplets in three dimensions. García’s team is experimenting with moving two drops together on a vertical surface to form a single sausage-shaped drop. “The reason for this is to make it easier to do protein separation,” García says. “It’s easier to cut a cylindrical drop than a round one.”

García says the new technology could provide the pharmaceutical and biotechnology industries with improved ways to screen new drugs. Others see promise for this technology in improving public safety and homeland security efforts.

Tom Picraux, chief scientist at the Center for Integrated Nanotechnologies at Los Alamos National Laboratory, believes the method could aid in more quickly detecting and analyzing dangerous chemicals if they were intentionally introduced into a public environment. For example, he envisions detectors being installed at building entrances and exits. Because of their low power requirements, these detectors could be battery powered. The technology could also improve monitoring systems in factories and other industrial operations where potentially hazardous chemicals are in use.

Hayes says superhydrophobics are also of interest to people doing ship design and working with piping. “The military is specifically interested in how this technology could be used to reduce the turbulence signature—bioluminescence—caused by ships’ propellers as they move through the ocean,” he says. Enemy aircraft can track a ship at night by following the trail of luminescence it leaves behind.

Researchers in the Department of Chemical and Biomolecular Engineering at the University of Illinois at Urbana–Champaign are currently building the first 3-D microfluidic devices. Using a device like an ink jet, the researchers extrude a very fine ink filament that is then patterned so as to form a matrix with continuous channels that run in all three dimensions. The matrix is encapsulated in a resin, the resin is heated, and the ink liquefies and is removed, leaving an open channel into which a magnetic element can be infused.

Jennifer Lewis, a professor of chemical and biomolecular engineering at the University of Illinois at Urbana–Champaign, anticipates revolutionary applications for such devices in the future. “We see the possibility of developing ‘self-healing’ materials using microvascular elements,” she says. The network of channels would essentially act as a circulatory system, carrying repair chemicals to damaged sites in a material.

Discrete magnetic microfluidics is not currently being used in commercial applications. García estimates the technology is probably five years from a first-generation commercial device. However, industry spokespersons definitely see uses for it. Gary Witting, an engineer and registered patent attorney in Scottsdale, Arizona, says, “There are a lot of potential applications of this technology to improve the lives of people.”

## Figures and Tables

**Figure:**